# The Mediator complex subunits MED25/PFT1 and MED8 are required for transcriptional responses to changes in cell wall arabinose composition and glucose treatment in *Arabidopsis thaliana*

**DOI:** 10.1186/s12870-015-0592-4

**Published:** 2015-09-05

**Authors:** Mathilde Seguela-Arnaud, Caroline Smith, Marcos Castellanos Uribe, Sean May, Harry Fischl, Neil McKenzie, Michael W. Bevan

**Affiliations:** Cell and Developmental Biology Department, John Innes Centre, Colney Lane, Norwich, NR4 7UH UK; INRA, Institut Jean-Pierre Bourgin, UMR1318, ERL CNRS 3559, Saclay Plant Sciences, RD10, 78000 Versailles, France; Nottingham Arabidopsis Stock Centre, University of Nottingham, School of Biosciences, Loughborough, LE12 5RD UK; Department of Biochemistry, University of Oxford, South Parks Rd, Oxford, OX1 3QU UK

## Abstract

**Background:**

Plant cell walls are dynamic structures involved in all aspects of plant growth, environmental interactions and defense responses, and are the most abundant renewable source of carbon-containing polymers on the planet. To balance rigidity and extensibility, the composition and integrity of cell wall components need to be tightly regulated, for example during cell elongation.

**Results:**

We show that mutations in the *MED25/PFT1* and *MED8* subunits of the Mediator transcription complex suppressed the sugar-hypersensitive hypocotyl elongation phenotype of the *hsr8-1* mutant, which has cell wall defects due to arabinose deficiency that do not permit normal cell elongation. This suppression occurred independently of light and jasmonic acid (JA) signaling. Gene expression analyses revealed that the expression of genes induced in *hsr8-1* that encode enzymes and proteins that are involved in cell expansion and cell wall strengthening is reduced in the *pft1-2* mutant line, and the expression of genes encoding transcription factors involved in reducing hypocotyl cell elongation, genes encoding cell wall associated enzymes and proteins is up-regulated in *pft1-2. PFT1* was also required for the expression of several glucose-induced genes, including those encoding cell wall components and enzymes, regulatory and enzymatic components of anthocyanin biosynthesis, and flavonoid and glucosinolate biosynthetic pathways.

**Conclusions:**

These results establish that MED25 and MED8 subunits of the Mediator transcriptional complex are required for the transcriptional regulation of genes involved in cell elongation and cell wall composition in response to defective cell walls and in sugar- responsive gene expression.

**Electronic supplementary material:**

The online version of this article (doi:10.1186/s12870-015-0592-4) contains supplementary material, which is available to authorized users.

## Background

Sugars are universal nutrients that provide carbon skeletons for energy production, storage and the synthesis of most metabolites. In plants, the main sink of carbon is the cell wall [1], a dynamic structure that provides both rigidity to support the plant and plasticity to allow cell growth. There is extensive knowledge of the enzymes involved in the synthesis and assembly of cell wall polysaccharides [2–4], but relatively little is known about how environmental stimuli and photosynthate availability contribute to cell wall formation during cell growth.

Sugars can act as both metabolic intermediates and as signaling molecules [5], and treatment of plants with sugars promotes growth. One mechanism linking sugar availability and growth promotion is the stimulation of auxin synthesis by exogenous sugars [6], which may indirectly influence cell wall formation by promoting cell elongation. Sugar levels may also link cell wall formation with the maintenance of turgor pressure. Mutations in a gene encoding a cell wall-associated kinase (WAK), which is required for normal cell expansion, also exhibited reduced vacuolar invertase activity [7]. This led to an increased dependence of seedlings on exogenous sugars for maintaining turgor and growth, and indicated that WAKs may be involved in maintaining the balance between turgor pressure, which drives cell expansion, and cell wall formation. A similar link between turgor and cell walls was shown by interrupting cellulose synthesis and observing that the resulting stress responses and distorted cells were rescued by osmotic support and sugar availability [8]. The interaction between sugar signaling and cell wall integrity control was also highlighted by the sugar hypersensitivity of several cell wall matrix structural mutants *mur4*, *mur1* and *mur3* [9]. The *hsr8-1* (*high sugar response 8-1*) allele of *MUR4*, which is defective in UDP-Arabinose synthesis, exhibits sugar hypersensitive gene expression and growth responses [9]. The *pleitropic regulatory locus1* (*prl1*) mutation was identified as a suppressor of *hsr8-1* sugar hypersensitivity phenotypes. *PRL1* (Pleiotropic Regulatory Locus 1) encodes a WD40 protein that is a component of a spliceosome complex, and *prl1* mutations have multiple complex phenotypes that include sugar hypersensitivity [10]. These findings suggest that impaired cell wall composition may be actively sensed, leading to transcriptional responses that modify cell wall composition and growth [11].

Recently, the existence of such transcriptional regulators controlling cell wall integrity and plant growth was demonstrated [12, 13]. The stunted growth and lignin deficiency of the lignin deficient mutant *ref8* was restored by the disruption of two subunits of the transcriptional regulatory complex Mediator, MED5a and MED5b. Here we show that the MED25/PFT1 (MEDIATOR25/PHYTOCHROME AND FLOWERING TIME 1) and MED8, two other subunits of the Mediator transcription complex, are able to suppress the sugar hypersensitive short hypocotyl and gene expression phenotypes of the *hsr8-1* mutant. We show that these Mediator subunits are required for the altered expression of a set of genes encoding cell wall components and biosynthetic activities in the *hsr8-1* mutant [9]. We show that one of these subunits, MED25/PFT1, is also required for the coordinated induction of several sugar-responsive genes, including those encoding cell wall modifying enzymes. These results suggest the MED25 and MED8 subunits of the Mediator complex have an integrating role by linking sugar responsive- and cell wall- gene expression.

## Results

### Identification of a novel suppressor of *hsr8-1* sugar hypersensitive growth

**Fig. 1:**
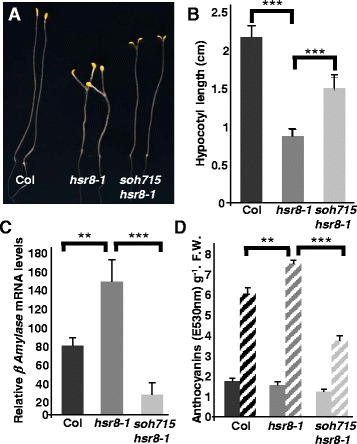
Identification of a suppressor of *hsr8-1* sugar-hypersensitive hypocotyl elongation in the dark. **a** Image of sugar hypersensitive hypocotyl elongation of Col, *hsr8-1* and *hsr8-1soh715* grown on 1 % glucose on vertical plates in the dark. **b** Quantitative measurements of hypocotyl lengths of Col, *hsr8-1* and *hsr8-1soh715*. Seedlings were grown vertically in the dark for 14 days on MS medium with 1 % Glucose. Errors bars represent SD (*n* > 30). ***, *p* < 0.001 comparing Col to *hsr8-1* and *hsr8-1* to *soh715 hsr8-1* (Student’s *t*- test). Data shown is representative of three independent experiments. **c** Quantitative Real-time PCR analysis of *β-Amylase* mRNA levels in Col, *hsr8-1* and the *hsr8-*1*soh715* repressor in response to glucose. Seedlings were grown on MS medium supplemented with 0.5 % glucose in constant light. After 7 days, seedlings were transferred 24 h in a MS glucose-free liquid medium and then treated for 6 h with 3 % MS medium containing 3 % glucose. Errors bars represent SD from three biological replicates. Data shown is representative of three independent experiments. **, *p* < 0.01 comparing Col to *hsr8-1*; ***, *p* < 0.001 comparing *hsr8-1* to *soh715 hsr8-1* (Student’s *t*- test). Relative transcript levels (RTL) were calculated using transcript levels of the reference gene *TUB6* (At5g12250). **d** Anthocyanin accumulation in response to glucose in Col, *hsr8-1* and *hsr8-1soh715*. Seedlings were grown in continuous light for 7 days on MS medium containing 1 % glucose (solid bars) or 3 % glucose (dashed bars). Errors bars represent SD from three biological replicates. **, *p* < 0.01 comparing Col to hsr8-1; ***, *p* < 0.001 comparing *hsr8-1* to *soh715 hsr8-1* (Student’s *t*- test). Data shown is representative of two independent experiments

The *high sugar response8-1* mutant, which has reduced cell wall arabinose [14], displays a range of sugar hypersensitivity phenotypes [9]. Among these, dark grown *hsr8-1* seedlings show reduced hypocotyl elongation in response to glucose in comparison to wild-type plants, and light-grown seedlings show hypersensitive sugar-regulated gene expression and anthocyanin content. To identify possible mechanisms linking altered cell wall composition and sugar responses, we screened for suppressors of the short hypocotyl phenotype of the *hsr8-1* mutant. We grew M2 seedlings of a fast neutron mutagenized *hsr8-1* population in the dark in the presence of glucose for 14 days and screened for individuals with longer hypocotyls. Eight suppressors of *hsr8-1* (*soh*) were isolated, several deletions were genetically mapped, and the *soh715hsr8-1* recessive mutant was selected for further analysis. Figures 1a and b show the intermediate hypocotyl length of the *soh715hsr8-1* suppressor mutant compared to wild- type Col and *hsr8-1*. The *hsr8-1* sugar hypersensitive phenotypes, increased β-amylase (*BAM*) mRNA accumulation and anthocyanin content were also suppressed in the *soh715hsr8-1* mutant, with *BAM* mRNA accumulation and anthocyanin content reduced in *hsr8-1* to lower levels than in wild-type plants (Fig. 1c and d). These results show that the *soh715* mutation suppresses *hsr8-1* sugar hypersensitive phenotypes.

### *soh715* is allelic to the *pft1-2* mutation

**Fig. 2:**
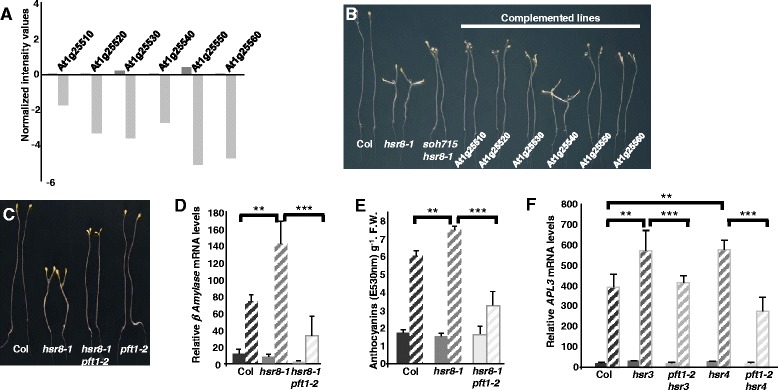
*hsr8-1* sugar hypersensitive phenotypes are suppressed by the *pft1-2* mutation. **a** Identification by microarray analysis of a cluster of six genes that are down regulated in the suppressor line *soh715hsr8-1* compared to *hsr8-1*. Values on the Y-axis are those obtained after normalization of the entire microarray data set. Dark grey bars and light grey bars represent values obtained for the *hsr8-1* mutant and the *hsr8-1soh715* suppressor line respectively. **b** Sugar hypersensitive dark development of Col, *hsr8-1, soh715hsr8-1* and *soh715hsr8-1* complemented with each of the 6 genes of the deletion. Seedlings were grown vertically in the dark for 14 days on MS medium containing 1 % Glucose. Only the genomic fragment containing the At1g25540 gene rescued the dark development phenotype. **c** Sugar hypersensitive dark development of Col, *hsr8-1,* the double mutant *hsr8-1pft1-2* and *pft1-2*. Seedlings were grown as described in (B) above. **d** Quantitative Real-time PCR analysis of *β-Amylase* mRNA levels in Col, *hsr8-1* and the double mutant *hsr8-1pft1-2* in response to glucose. Seedlings were grown on MS medium supplemented with 0.5 % glucose in constant light. After 7 days, the seedlings were transferred for 24 h to MS glucose-free liquid medium and then treated for 6 h with MS medium containing 3 % glucose. Errors bars represent SD from three biological replicates. Data shown is representative of three independent experiments. **, *p* < 0.01 comparing Col to *hsr8-1*; ***, *p* < 0.001 comparing *hsr8-1* to *hsr8-1 pft1-2* (Student’s *t*- test). Relative transcript levels (RTL) were calculated using transcript levels of the reference gene *TUB6* (At5g12250). **e** Anthocyanin accumulation in response to glucose in Col, *hsr8-1* and the double mutant *hsr8-1pft1-2*. Seedlings were grown in continuous light for 7 days on MS medium containing 1 % glucose (solid bars) or 3 % glucose (dashed bars). Errors bars represent SD from three biological replicates. Data shown is representative of two independent experiments. **, *p* < 0.01 comparing Col to hsr8-1; ***, *p* < 0.001 comparing *hsr8-1* to *hsr8-1 pft1-1* (Student’s *t*- test). **f** Quantitative Real-time PCR analysis of the sugar-responsive *APL3* gene mRNA levels in Col, *hsr3*, *pft1-2hsr3, hsr4*, *pft1-2hsr4* and *pft1-2* in response to glucose*.* Hsr3 and hsr4 are sugar-hypersensitive mutations in subunits of the ARP2/3 complex [18]. Seedlings were grown on MS medium supplemented with 0.5 % glucose in constant light. After 7 days, the seedlings were transferred to glucose-free liquid MS medium for 24 h and then treated for 6 h with MS medium containing either 0 % glucose (solid bars) or 3 % glucose (dashed bars). Errors bars represent SD from three biological replicates. **, *p* < 0.01 comparing Col to *hsr3* and Col to *hsr4*; ***, *p* < 0.001 comparing *hsr3* to *hsr3 pft1-2* and *hsr4* to *hsr4 pft1-2* (Student’s *t*- test). Relative transcript levels (RTL) were calculated using transcript levels of the reference gene *TUB6* (At5g12250)

To map the *soh715* locus in the Columbia ecotype, the double mutant was crossed with wild-type Landsberg *erecta*. To isolate *soh715hsr8-1* double mutant plants, long hypocotyl plants were selected in the F2 population and genotyped to identify *hsr8-1* homozygous plants. Double *soh715hsr8-1* mutants comprised 1/4^th^ of the segregating population instead of the expected 1/16^th^. Preliminary genetic analysis (data not shown) showed the *soh715* locus mapped to a region of chromosome 1 where *HSR8* also maps, confirming that the *soh715* and *hsr8-1* mutations may be genetically linked. A transcript-based cloning approach [15] was then used to identify deletions in the mapped region. Gene expression in *hsr8-1* and *soh175hsr8-1* seedlings was assessed using the ATH1 Gene Chip. Comparison of gene expression levels revealed that 6 consecutive genes on chromosome 1 showed strongly reduced RNA levels in *soh715hsr8-1* compared to *hsr8-1* (At1g25510, At1g25520, At1g25530, At1g25540, At1g25550, At1g25560; Fig. 2a). This result, taken together with the preliminary genetic mapping data, indicated that a deletion encompassing 6 genes on chromosome 1 suppressed the *hsr8-1* phenotype. To identify the gene(s) involved, we complemented the *soh715hsr8-1* mutant background with 6 genomic fragments, each containing one gene and flanking regions in the deleted locus. Only the genomic fragment containing At1g25540 restored the *hsr8-1* short hypocotyl phenotype (Fig. 2b). The suppression of *hsr8-1* dark development phenotype in the *soh715hsr8-1* mutant is therefore caused by the deletion of At1g25540, encoding the MED25/PFT1 protein [16, 17]. To confirm this observation a double mutant between *hsr8-1* and a loss-of function T-DNA insertion allele in At1g25540 called *pft1-2* was analysed. When grown in the dark in the presence of glucose, the *hsr8-1pft2-1* double mutant displayed the same intermediate hypocotyl length as *soh715hsr8-1* (Fig. 2c). Increased accumulation of *BAM* transcripts and anthocyanins in *hsr8-1* in response to glucose treatment was suppressed by the *pft1-2* mutation (Fig. 2d and e). Figure 2f shows that *pft1-2* also suppresses elevated glucose- responsive *APL3* expression in the glucose hypersensitive mutants *hsr3* [18] and *hsr4*, which is a mis-sense mutation in the ARP3 subunit of the Arp2/3 complex(unpublished data). Therefore loss of *PFT1* gene function suppressed the *hsr8-1* hypocotyl cell elongation defect and sugar hypersensitive gene expression.

### Cell wall composition is altered in *pft1-2*

**Fig. 3:**
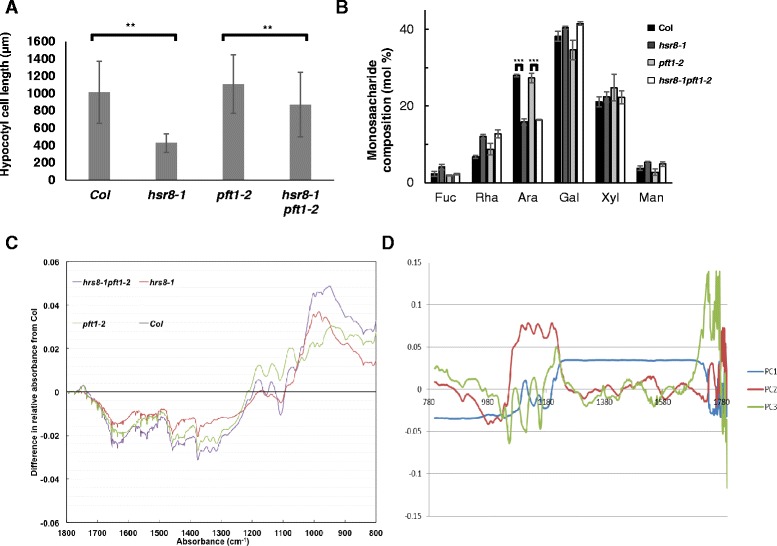
Comparison of cell elongation and cell wall composition in Col, *hsr8-1*, *pft1-2* and *hsr8-1pft1-2*. **a** Hypocotyl cell length was measured from scanning electron micrograph images. *n* = 10 cells each from 5 hypocotyls. **, *p* < 0.01 comparing *hsr8-1* to Col, and *hsr8-1* to *pft1-2* and *hsr8-1 pft1-2* (Student’s *t*- test). **b** Monosaccharide composition of cell wall material isolated from 14 day old light grown seedlings (5 biological replicates). Fuc fucose; Rha rhamnose; Ara arabinose; Xyl xylose; Man mannose. . ***, *p* < 0.001 comparing arabinose levels in Col to *hsr8-1*, and *pft1-2* to *hsr8-1 pft1-2* (Student’s *t*- test). **c** Compositional analysis of cell wall material isolated from dark grown hypocotyl tissues using FTIR. The data are represented as differences in relative absorbance from wild-type Col. **d** Principal Components Analyses of FTIR data. Score loadings of PC1, PC2 and PC3 are plotted against the range of wavelengths to show the major variance

Figure 3a shows that reduced hypocotyl elongation in *hsr8-1*, and its suppression by *pft1-2*, was due to changes in cell length and not in cell number. The suppression of the short hypocotyl phenotype in *hsr8-1* by *pft1-2* was not due to changes in cell wall monosaccharide composition, as the *hsr8-1pft1-2* double mutant had the same reduced levels of arabinose as *hsr8-1* (Fig. 3b). Additional analyses of cell wall composition of hypocotyls of dark-grown Col, *hsr8-1, hsr8-1pft1-2* and *pft1-2* mutants seedlings were conducted using Fourier Transform InfraRed spectroscopy (FTIR) [19]. Figure 3c shows difference spectra relative to wild-type Col, and Principle Components Analysis (PCA) identified three principle components when mapped as score loadings (Fig. 3d). PC1 explained ~80 % of the variation in cell wall composition between genotypes, showing very broad variation across the spectra with positive loadings between 800 and 1200 cm-1 and depletion at 1200-1800 cm-1 relative to Columbia. Although PC2 and PC3 explained less variation (~15 and 4 % respectively), these principle components identified variation in more specific spectra between genotypes. In PC2, the positive loading between 1120 and 1097 cm-1 may reflect variation in xyloglucan and pectin respectively between genotypes [19]. PC3 identifies positive loadings between 1660 and 1776 cm-1, possibly reflecting differences in waxes and phenolic composition [19]. Additional file 1: Figure S1 are scatter plots comparing PC1, PC2 and PC2 between the mutants. There were significant differences between each of the genotypes for each of the three PCs.

### Suppression of *hsr8-1* by *pft1-2* is not dependent on the *phyA*, *phyB* or jasmonate pathways

**Fig. 4:**
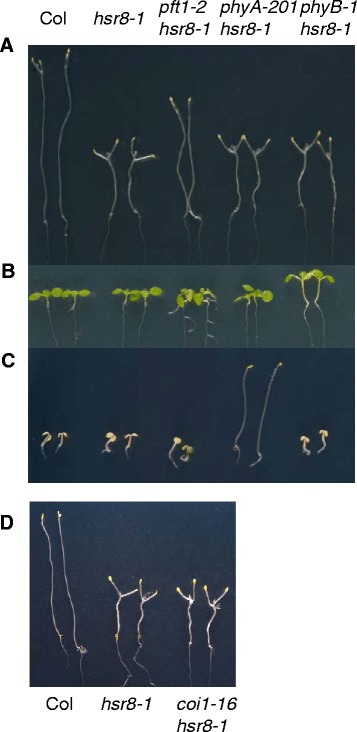
*PFT1* acts independently of *phyA* and *phyB* and the jasmonate response pathway in the suppression of the *hsr8-1* hypocotyl elongation phenotype. **a** Sugar-hypersensitive dark development of Col, *hsr8-1*, *hsr8-1pft1-2*, *phyA-201hsr8-1*, *phyB-1hsr8-1* mutants. Seedlings were grown vertically in the dark for 14 days on MS medium containing 1 % glucose. **b** Hypocotyl phenotypes of Col, *hsr8-1*, *hsr8-1pft1-2*, *phyA-201hsr8-1*, *phyB-1hsr8-1* mutants grown in white light. Seedlings were grown 7 days on MS sugar free medium under constant white light. **c** Hypocotyl phenotypes in far-red light of Col, *hsr8-1*, *hsr8-1pft1-2*, *phyA-201hsr8-1*, *phyB-1hsr8-1* mutants. Seedlings were grown 4 days on MS sugar free medium under constant far-red light. **d** Sugar hypersensitive hypocotyl elongation of Col, *hsr8-1*, *coi1-16hsr8-1* mutants. Seedlings were grown as in (**a**)

MED25/PFT1 was first identified as a positive regulator of flowering in response to sub-optimal light conditions, and *pft1* mutants display slightly longer hypocotyls in far red light and a late flowering phenotype in long days [16]. As mutants with longer hypocotyls were identified in our screen, and because phyA has been implicated in sugar responses [20], we assessed the role of phytochrome signalling pathways in the suppression of *hsr8-1* sugar hypersensitivity. Neither the *phyA-201* [21] nor the *phyB-1* [22] mutations suppressed the *hsr8-1* dark development phenotype (Fig. 4a). Seedlings were also grown under constant white light (Fig. 4b) and constant far-red light (Fig. 4c) to confirm that the *phyA-201hsr8-1 *and *phyB-1hsr8-1* double mutants displayed characteristic *phyA* and *phyB* phenotypes, unlike the *pft1-2hsr8-1* double mutant.

*PFT1* is a regulator of the jasmonate (JA) signalling pathway [23]. As cell wall defects can trigger defence responses through the jasmonate signalling pathway [24, 25], we tested whether JA-dependent defence responses were activated in *hsr8-1* and if *pft1-2* suppressed *hsr8-1* sugar hypersensitivity through the JA signalling pathway. Expression of *VSP1*, *VSP2* and *ERF1*, which are strongly up regulated by JA, was not up- regulated in *hsr8-1* compared to Col in dark-grown seedlings (Additional file 1: Figure S2). This showed that the JA pathway was not induced in *hsr8-1* in response to its cell wall defect. Crosses to the JA- insensitive mutant *coi1-16* [26] confirmed this; the *coi1-16hsr8-1* double mutant had the same short hypocotyl phenotype as *hsr8-1* (Fig. 4d). Therefore suppression of the *hsr8-1* short hypocotyl phenotype by *pft1-2* is independent of its role in the JA and phytochrome signalling pathways.

### Microarray analysis of *pft1-2-*dependent gene expression

**Fig. 5:**
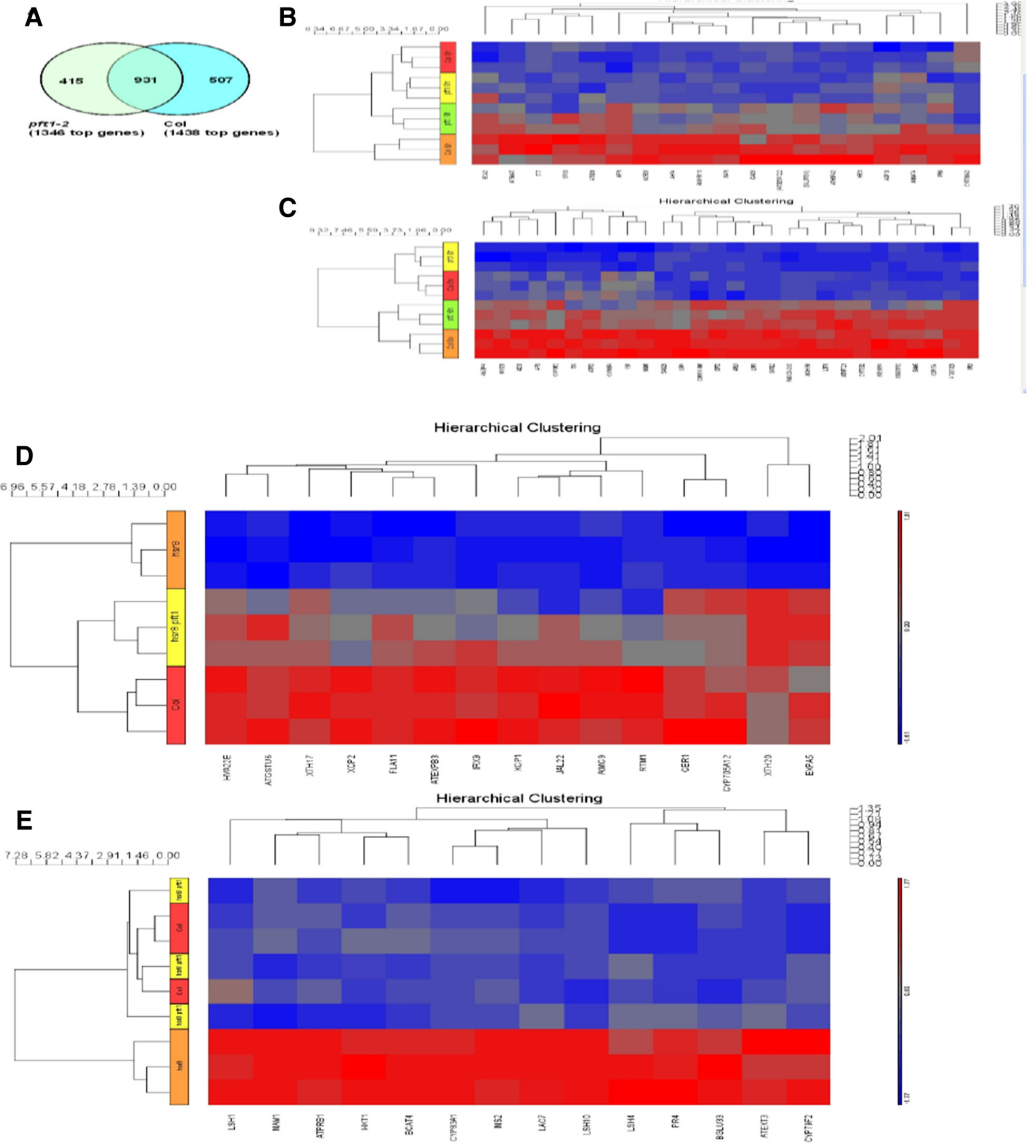
Microarray analyses of gene expression in Col, *hsr8-1*, *pft1-2* and *hsr8-1pft1-2* seedlings. **a** Venn diagram of glucose- induced genes in Col and *pft1-2.* **b** Hierarchical clustering of 19 genes showing no induction in response to glucose in *pft1-2* compared to Col. **c** Hierarchical clustering of 28 genes showing reduced induction in response to glucose in *pft1-2* compared to Col. **d** Hierarchical clustering of 15 genes that were down- regulated in *hsr8-1* compared to Col, and up- regulated in *hsr8-1pft1-2* compared to *hsr8-1* in dark grown seedlings. These genes require *PFT1* for repression in response *to hsr8-1*. **e** Hierarchical clustering of 14 genes that were up- regulated in *hsr8-1* compared to Col, and down- regulated in *hsr8-1pft1-2* compared to *hsr8-1* in dark grown seedlings. These genes require *PFT1* for induction in response to *hsr8-1*

*PFT1* encodes subunit 25 of the Mediator complex, a conserved regulator of transcription in eukaryotes [17, 27]. We therefore assessed the extent to which *PFT1* controls gene expression in response to glucose in light grown seedlings, and also how it controls gene expression during dark development in Col and *hsr8-1* genetic backgrounds. For glucose-responsive gene expression, three independent replicates of *pft1-2* and wild-type light-grown 7 day old seedlings were collected 6 h after 3 % glucose or 0 % glucose treatment. Two-way ANOVA (Analysis of Variance) (Additional file 2) revealed that 1438 genes were differentially expressed in response to 3 % glucose in Col and 1346 genes in *pft1-2* (Fig. 5a), of which 931 genes were differentially expressed in response to glucose in both genotypes. A total of 92 genes had fold changes between -2 and +2, and 47 genes were induced >2 fold by glucose in wild-type Col. Nineteen of these showed no significant glucose- dependent induction in *pft1-2* and 28 showed strongly reduced glucose- dependent induction in *pft1-2* (Fig. 5b and c). The expression of five general categories of genes were either completely or partially dependent on *PFT1* for increased expression in response to glucose. Expression of six genes involved in the regulation, biosynthesis and transport of anthocyanins required *PFT1*, including the central regulator *MYB75/PAP1* [28]. Five genes encoding uptake transporters of nitrate, phosphate and sulphate, and the phosphate uptake regulator *SPX3*, also required *PFT1* for increased expression in response to glucose [29, 30]. Seven genes encoding enzymes (primarily cytochrome P540s) in the biosynthesis of glucosinolates required *PFT1* for their expression in response to glucose. Of the four MYB transcription factor genes involved in regulating glucosinolate (GSL) biosynthesis [31, 32], *MYB28/HAG1* required *PFT1* for increased expression. Thirteen genes encoding a wide variety of stress responsive genes required *PFT1* for expression in response to glucose. These include two *COR* (COld Regulated)-related genes, *NCED3* involved in ABA biosynthesis, the *AFP1* gene encoding an ABI5 binding protein, the heat-shock transcription factor *HSFA2*, a *PIRIN* gene involved in ABA signaling, *bZIP44* involved in regulating proline dehydrogenase, and *ECA2* encoding an ER Ca^2+^ transporter involved in stress responses. Finally, several genes encoding proteins involved in cell expansion required *PFT1* for their glucose-responsive expression, including two Lipid Transfer Proteins (LTPs) involved in membrane modifications, and Expansin 4, which is involved in cell wall extension [33, 34].

To confirm and extend these microarray analyses, we measured the influence of *PFT1* on the expression of a small set of well- characterised glucose-responsive genes identified previously in microarray experiments [35]. Q-RTPCR analysis showed that the glucose-induced genes *APL3*, *BAM*, *GBSS1*, *GPT2* and *PDC1* all had reduced expression in *pft1-2* (Additional file 1: Figure S3A-S3E), and confirmed the microarray data showing reduced expression of *APL3* and *BAM*. The expression of genes encoding enzymes in the anthocyanin synthesis pathway (*FLS*, *CHS* and *TT6*) was also assessed by Q-RTPCR, and they all showed reduced expression in *pft1-2* (Additional file 1: Figure S3F-3H). Finally, anthocyanin levels were reduced to 45 % of wild-type levels in *pft1-2* (Additional file 1: Figure S2I), confirming the important role of PFT1 in the expression of regulators and enzymes of anthocyanin synthesis.

Gene expression in 14-day old dark-grown seedlings of Col*, hsr8-1, hsr8-1pft1-2* and *pft1-2* was measured in three independent RNA samples using microarray analysis. Two-way ANOVA analysis (Additional file 3) identified 76 genes that were ≥2 fold up- or down- regulated in *hsr8-1* compared to Col, and 44 genes were differently regulated between *hsr8-1* and *hsr8-1pft1-2.* There were 29 genes in common that were differentially regulated in *hsr8-1* vs Col and *hsr8-1* vs *hsr8-1pft1-2*. These genes were clustered according to their expression patterns (Fig. 5d and e). Of the 15 genes that were significantly down- regulated in *hsr8-1* compared to Col and up- regulated in *pft1-2hsr8-1* compared to *hsr8-1* (that is, requiring PFT1 for repressing their expression in *hsr8-1*), 10 encode proteins involved in cell wall formation, cuticle formation and cell expansion. These include *XTH17* and *XTH20*, encoding xyloglucan endo-transglycosidase/hydrolase enzymes that cleave and re-arrange xyloglucans [36]), and *IRX9* encodes a xylosyl transferase involved in xylan synthesis [37]. *EXPB5* and *EXP5* encode the cell wall proteins expansin B3 and expansin 5 that promote cell wall expansion, *CER1* encodes an enzyme of cutin formation, *FLA11* encodes a fascilin-type arabinogalactan protein involved in cell adhesion, *RTM* encodes a mannose-binding lectin, and *JAL22* encodes an ER-Golgi transporter that may be involved in the transport of cell wall components to the plasma membrane. The expression of three genes encoding peptidases involved in programmed cell death in xylem, *XCP1, XCP2* and the metacaspase-encoding gene *MC9* was reduced in *hsr8-1* and increased in *hsr8-1pft1-2*. Similarly the expression of two stress-induced genes, *HVA22* and *GSTU6* encoding glutathione-S-transferase, was reduced in *hsr8-1* compared to Col, and increased in *hsr8-1pft1-2* compared to *hsr8-1*.

The expression of a diverse set of 14 genes was increased in *hsr8-1* compared to Col and decreased in *hsr8-1pft1-2* compared to *hsr8-1* (Fig. 5e). These genes required PFT1 for their increased expression in *hsr8-1*. Three members of the light-dependent short hypocotyl (*LHS1, 4* and *10*) gene family, encoding conserved nuclear proteins of the ALOG (Arabidopsis LSH1 and Oryza G1) family of transcription factors [38], and five genes encoding enzymes of methionine- and aliphatic glucosinolate biosynthesis [31] required PFT1 for increased expression in *hsr8-1*. Three genes encoding the cell wall hydroxyproline rich glycoprotein Extensin 3, laccase involved in lignin biosynthesis, and β-glucosidase 33 also required *PFT1* for increased expression in *hsr8-1* compared to Col. Finally *HKT1*, encoding a protein involved in sodium retrieval from xylem, was expressed in a similar pattern.

To extend these analyses, q-RTPCR analyses of genes encoding cell wall components and enzymes with increased expression in *hsr8-1* compared to Col [9] was carried out in the double mutant *pft1-2hsr8-1*. These analyses showed that increased expression in *hsr8-1* of *EXT3*, *EXT4*, encoding cell wall glycoproteins, and *PME17* and *PME41*, encoding pectin methylesterases, was reduced in *hsr8-1pft2-1* (Additional file 1: Figure S4), confirming the increased expression of *EXT3* seen in microarray data and extending the range of cell wall-related genes requiring *PFT1* in *hsr8-1*.

### MED8 is also required for the expression of selected genes encoding cell wall components but is a repressor of glucose-induced gene expression

**Fig. 6:**
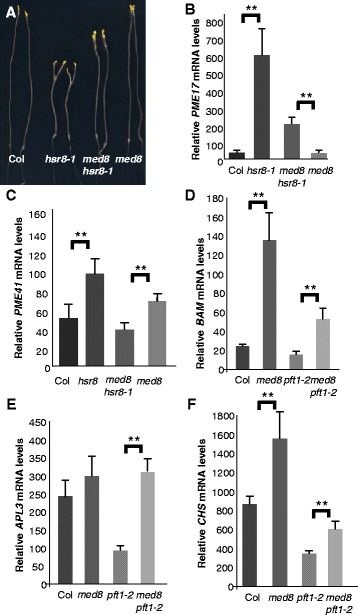
The MED8 subunit plays a role in sugar responsive growth and gene expression. **a** Sugar hypersensitive dark development of Col, *hsr8-1, med8hsr8-1* and *med8* mutants. Seedlings were grown vertically in the dark for 14 days on MS medium containing 1 % Glucose. **b** and **c** Quantitative Real-time PCR analysis of mRNA levels of cell wall modifying encoding genes PME17 and *AtPME41* in Col, *hsr8-1*, *med8hsr8-1* and *med8.* Seedlings are grown as described in (**a**) above. Errors bars represent SD from three biological replicates. **, *p* < 0.01 comparing Col to *hsr8-1*, and *med8 hsr8-1* to *med8* (Student’s *t*- test). Relative transcript levels (RTL) were calculated relative to the transcript level of the reference gene *TUB6* (At5g12250). **d** to **f** Quantitative Real-time PCR analysis of *BAM, APL3,* and *CHS* mRNA levels in Col, *med8, pft1-2* and the double mutant *med8pft1-2* in response to glucose. Seedlings were grown on MS medium supplemented with 0.5 % glucose in constant light. After 7 days, the seedlings were transferred to glucose-free liquid MS medium for 24 h and then treated for 6 h with 3 % Glucose. Errors bars represent SD from three biological replicates. **, *p* < 0.01 comparing Col to *med8* and *pft1-2* to *med8 pft1-2* (D); **, *p* < 0.01 comparing *pft1-2* to *med8 pft1-2* (E); **, *p* < 0.01 comparing Col to *med8* and *pft1-2* to *med8 pft1-2* (F) (Student’s *t*- test). Relative transcript levels (RTL) were calculated using transcript levels of the reference gene *TUB6* (At5g12250)

The Mediator complex in *Arabidopsis* is composed of at least 27 subunits [17], therefore we examined other subunits in addition to PFT1/MED25 for a potential role in sugar- and cell elongation- mediated gene expression. *med8* mutants exhibited similar phenotypes to *pft1-2* with respect to pathogen responses, flowering time and organ size [39, 40]. Furthermore, the yeast homolog of MED8 was shown to be involved in sugar signalling [41]. To test the involvement of MED8, *hsr8-1* was crossed with a loss of function T-DNA insertion *med8* mutant, and hypocotyl length in dark developed seedlings was analysed. As shown in Fig. 6a, *med8* suppresses the *hsr8-1* short hypocotyl phenotype to the same extend as *pft1-2* (compare with Fi. 1a). We therefore measured expression of the same set of four *PFT1*-responsive cell wall- related genes shown in Additional file 1: Figure S4 in *hsr8-1* and *med8hsr8-1* in dark grown seedlings. Figure 6b and c shows that expression of two of these four, *PME17* and *PME41*, was substantially reduced in *med8hsr8-1* compared to *hsr8-1*. Analysis of glucose- induced gene expression in light- grown *med8* seedlings showed an opposite effect to that observed in the *pft1-2* mutant: the *med8* mutant significantly enhances expression of three genes with well-characterised responses to glucose, *BAM*, *APL3* and *CHS* (Fig. 6d, e and f). This increase was consistently less in the double mutant *med8pft1-2* in the analysed genes, suggesting that MED8 and PFT1 have opposing effects on glucose-induced gene expression.

## Discussion

A genetic screen for mutants that suppressed the short hypocotyl phenotype of dark-grown *hsr8-1* seedlings identified eight *soh* mutants. One mutant, *soh715hsr8-1*, had an intermediate hypocotyl length when grown in the dark (Fig. 1a and b) and also suppressed hypersensitive responses to glucose as assessed by gene expression and anthocyanin accumulation (Fig. 1c and d). The elongated cotyledonary petioles seen in *hsr8-1* [9] were also partly suppressed by the *soh715* locus (Figs. 1a and 2b), but the main phenotype studied was the large difference in hypocotyl elongation, which was shown to be due to increased cell elongation (Fig. 3a). *soh715* was identified as *PFT1* (Fig. 2b) [16], encoding a subunit of the Mediator transcription complex [27] and confirmed by the double mutant *hsr8-1pft1-2* (Fig. 2c), which was used in subsequent analyses. *pft1* mutants exhibit longer hypocotyls in response to phytochrome-mediated signals [16, 42], increasing signalling downstream of PhyA and genetically interact with *HY5* [43]. Figure 4 shows that the dark-development phenotypes of *hsr8-1* were not dependent on phyA or PhyB, and *hsr8-1* did not significantly influence white- and far- red light responses. Furthermore, the dark development phenotypes of *hsr8-1* were not dependent on jasmonate responses [23]. We concluded that PFT1-mediated suppression of reduced hypocotyl elongation in *hsr8-1* was not dependent on PFT1 functioning as part of phytochrome- and JA- mediated responses, suggesting PFT1 functions through a independent mechanism(s) to reduce hypocotyl cell elongation during dark development of arabinose-deficient mutants.

The Mediator complex is a functionally conserved regulator of gene expression composed of approximately 30 subunits, forming a complex that docks transcription factors bound to enhancers with core promoter components such as RNA polymerase II [17, 27, 44]. Mediator also has a structural role in chromatin by forming a complex with cohesin that is associated with chromatin looping of promoters [45]. PFT1/MED25 forms part of the tail region of the complex that interacts with transcription factors, while MED8 is part of the head region interacting with core promoter components [27]. In metazoans, many diverse transcriptional regulatory networks converge on Mediator [27], with increasing evidence that different transcription factors interact with different subunits of the tail region. In plants, *PFT1/MED25* and *MED8* are required for expression of JA-responsive and fungal resistance genes [23, 46] and have antagonistic effects on organ size [40, 47]. *PFT1/MED25* is also required for drought-responsive gene expression [42] and is also directly involved in light responses and promoting flowering [16, 43, 48]. The Mediator subunits MED5a/5b repress expression of a set of phenylpropanoid and lignin biosynthetic genes [12, 13], and it was suggested that MED5a/5b may play a direct role in relieving growth repression caused by the phenylpropanoid mutant *ref8-1* through a cell wall sensing pathway.

Cluster analyses were conducted to identify two sets of genes in dark grown seedlings that were differentially regulated in *hsr8-1* compared to Col and in *pft1hsr8-1* compared to *hr8-1*. These sets comprise genes that required *PFT1* for increased or decreased expression in *hsr8-1* dark grown seedlings. Of the 15 genes with reduced expression in *hsr8-1* compared to Col, and increased expression in *hsr8-1pft1-2* compared to *hsr8-1*, ten encoded proteins involved in cell wall formation (Fig. 6a). Their expression profile shows that the expression of these genes is actively reduced in arabinose- deficient cell walls by PFT1, where they may limit cell wall expansion and/or compensate for altered cell wall composition. Among these are genes for xyloglucan chain modification (XTH17 and XTH20) [36, 49], and XTH17 which has xyloglucan endotransferase- hydrolase activity [50] involved in wall strengthening and expansion in response to shade cues [51, 52]. Expression of genes encoding expansins 5 and B3 was also repressed by PFT1 in *hsr8-1*. These cell wall proteins promote cell wall extensibility, possibly by loosening xyloglucan-cellulose interactions [53].

Fourteen genes encoding regulatory proteins, biosynthetic enzymes and the cell wall protein Extensin 3 had significantly elevated expression in *hsr8-1* compared to Col, and reduced expression in *hsr8-1pft1-2* compared to *hsr8-1* (Fig. 5d). The expression of three genes encoding LSH1, 4 and 10, members of the ALOG family of transcriptional regulators, was coordinately increased in *hsr8-1* in a *PFT1*-dependent pattern. Over-expression of *LHS1* led to reduced hypocotyl cell elongation [38], suggesting that PFT1-mediated expression of *LSH* family members may directly reduce hypocotyl cell elongation in *hsr8-1*. The increased expression of *Extensin 3* and *Extensin 4* in *hsr8-1* (Additional file 1: Figure S3) was dependent on PFT1, suggesting that the deficient cell walls in *hsr8-1* mutants may be strengthened by extensins, and that the reduced expression of *Extensin 3* and *Extensin 4* in *hsr8-1pft1-2* may contribute to increased cell wall extensibility associated with cell elongation.

In Col plants with normal cell walls, *PFT1* was required for the increased expression of seven genes encoding proteins that are involved in cell wall extension and cell elongation in response to high glucose levels: *Expansin 4* encodes a protein that loosens the wall by disrupting hydrogen bonds between cellulose and xyloglucan hemicelluloses [34] and *LTP3* and *LTP4* encode proteins implicated in cell membrane deposition and cell wall loosening [33]. PIF4 and PIF5 activate *LTP3* and *Expansin B1* gene expression and promote cell elongation [54], and PhyB negatively regulates this in the light. This is consistent with the known role of PFT1 in PhyB responses [16, 43] and suggests PIF4 and PIF5 may function in concert with PFT1 to promote cell elongation in response to light and glucose cues by activating *LTP* and *Expansin* gene expression.

Glucose levels strongly influence plant growth, and a key feature of glucose-mediated transcriptional responses involves the rapid coordinated expression of genes encoding enzymes and transporters involved in nutrient acquisition and the synthesis of secondary products and the co-expression of genes involved in ABA responses [35, 55]. Microarray analyses identified diverse classes of genes whose glucose-induced expression was fully or partly dependent on *PFT1/MED25*. These genes encoded cell wall- and cell expansion- related proteins, regulatory proteins and enzymes of anthocyanin, flavonoid and glucosinolate biosynthesis, regulators and transporters involved in nutrient uptake, ABA signaling and biosynthetic proteins, and a variety of stress-responsive proteins (Fig. 5b and c). Seven genes encoding enzymes and regulatory proteins in the biosynthesis of glucosinolates [32] required *PFT1* for increased expression in response to glucose. In the *hsr8-1* mutant *PFT1* was also required for the expression of five genes encoding enzymes of glucosinolate synthesis, with *MAM1* commonly regulated by glucose. The function of glucosinolate production in *hsr8-1* is not known, but the independence of PFT1-mediated *hsr8-1* phenotypes on JA indicates that stress responses may not be involved [23]. Notably, glucose-induced expression of *MYB75*, encoding a key anthocyanin pathway regulator [28] was completely dependent on *PFT1*. Recently MED5a and 5b have been shown to repress phenylpropanoid pathway gene expression [12, 13], establishing the central role of Mediator in integrating biosynthetic capacity in response to increased carbon supplies. Finally the *PFT1*- dependent expression of genes encoding nitrate, phosphate and sulphate transporters [56], and the phosphate uptake regulator *SPX3*, further demonstrate an important coordinating role for PFT1/MED25 in balancing nutrient supplies and carbon availability.

Reduced PFT1 function did not reconstitute wild-type cell wall arabinose content in *hsr8-1*, as shown by cell wall monosaccharide analyses (Fig. 3b), probably because *hsr8-1* is a loss of function allele of *MUR4*, which encodes the only known enzyme of UDP-arabinose synthesis in Arabidopsis [14]. Only large reductions in cell wall arabinose and fucose led to reduced hypocotyl elongation in the dark [9], which was rescued by low concentrations of borate. Borate cross-links rhamnogalacturonan II and is thought strengthen the cell wall, suggesting changes in cell wall composition and structure lead to reduced elongation in *hsr8-1* [9]. Analyses of cell wall polysaccharides using FTIR spectra of cell wall material from dark-developing hypocotyls showed complex quantitative changes in absorbance spectra in *hsr8-1* compared to wild-type Col, and in *hsr8-1pft1-2* compared to *pft1-2* and Col (Fig. 3c and d). Although there were significant differences between genotypes the major component of these differences showed variation across a broad range of wavelengths that precluded identification of specific polysaccharides with altered levels.

## Conclusions

Our analyses demonstrate a central role MED25 and MED8 subunits of the Arabidopsis Mediator complex in transcriptional responses involved in cell elongation, multiple biosynthetic pathways, stress responses, and nutrient acquisition in response to altered carbon availability.

## Methods

### Plant material and growth conditions

All experiments were carried out in the Columbia genetic background. The *hsr8-1, hsr3 and hsr4* mutants were isolated as previously described (Li et al. [9]; Baier et al. [57]). The suppressor mutants were isolated from an *hsr8-1* fast neutron mutagenized population (seeds were irradiated with 30-40 grays at the HAS KFKI-Atomic Energy Research Institute, Hungary). Plants containing T-DNA insertions in *PFT1* (SALK_129555), termed *pft1-2*, and *MED8* (SALK_592406), termed *med8*, were obtained from The European Arabidopsis Stock Centre (NASC, University of Nottingham, United Kingdom). Seeds were surface sterilized and sown on Murashige and Skoog (MS) medium containing 0.9 % agar and different glucose concentrations. Seeds were then stratified for 3 days at 4 °C and then grown in continuous light at 22 °C. For dark development experiments, seeds were grown on MS medium containing 1 % glucose, exposed to light for 8 h and then grown vertically in complete darkness for 2 weeks. For glucose treatment experiments, seedlings were grown on MS medium containing 0.5 % glucose for 7 days and transferred in MS liquid medium without glucose. After 24 h, the medium was changed to MS medium containing 3 % glucose and seedlings were collected 6 h later. For anthocyanin measurements, seedlings were grown on solid MS medium containing 1 or 3 % glucose for 7 days.

### Genetic screen and cloning of the *soh715* mutation

3200 M2 mutagenized lines were screened individually for increased hypocotyl length, in comparison to *hsr8-1*, when grown in the dark with 1 % glucose. Potential suppressor mutants were transferred to soil and the M3 progeny was rescreened. The *soh715* mutation was mapped by crossing to Landsberg *erecta* and F2 seeds were screened for long hypocotyls as described above and subsequently genotyped for homozygous *hsr8-1* mutation using the LightCycler®480 System and Hybprobes® technology (Roche Applied Science and TIB MOLBIOL GmbH). These plants were assumed to be homozygous for the *soh715* mutation and used for initial mapping of the mutation. Once the approximate location of the mutation was determined, total RNA of *hsr8-1* and *soh715hsr8-1* were extracted using the RNeasy plant mini kit (QIAGEN) and used for Affymetrix GeneChip array expression profiling to identify deleted genomic regions (Affymetrix, Santa Clara, CA, USA). We identified a cluster of six consecutive down-regulated genes in the *soh715hsr8-1*-mapped region on chromosome 1 that may be due to a deletion. The *soh715hsr8-1* mutant was transformed with genomic fragments containing the sequence of the six genes in the deletion. Additional file 4: Table S3 describes the primers used for cloning genomic fragments using the TOPO® XL PCR Cloning kit (Invitrogen). Cloned genes were subcloned in the pCAMBIA1300 binary vector using the *ApaI* restriction site and used for *Agrobacterium*-mediated transformation. Transgenic T1 plants were screened on 30 μg/mL hygromycin, and complementation of the *soh715hsr8-1* long hypocotyl phenotype was assessed in the T2 generation.

### Hypocotyl and anthocyanin measurements

Hypocotyl length was measured from 14-day old dark grown seedlings (*n* = 30) by scanning plates and using ImageJ software (http://rsb.info.nih.gov/ij/). Anthocyanins were extracted and quantified as described in [57].

### Cell wall analysis

Cell walls were prepared from frozen samples by boiling in 96 % ethanol for 10mins, homogenisation, repeated methanol:chloroform (2:3 v/v) extraction, 80 % ethanol extraction and dehydration in 96 % ethanol before drying at room temperature. Aliquots of 50 μg were dried, treated with 2 M TFA (trifuoroacetic acid) for 1 h at 120 °C, and then dried again. Samples were then resuspended in 5 % (v/v) acetonitrile and injected into an M-Scan High Performance Anion Exchange Chromatography system with Pulsed Amperometric Detection (HPAEC-PAD). Monosaccharides were detected using standards and values expressed as mole %. Fourier transform Infrared absorbance spectra were collected from 800 to 4000 cm^−1^ using a Biorad FTS 175C spectrophotometer. Hypocotyl material was ground and clamped against the diamond element. Two spectra from three biological replicates were obtained. Principle Components Analyses were conducted using Genstat version 15.

### Gene expression

Total RNA was extracted and DNase treated using the RNeasy Plant mini kit (QIAGEN). 2 μg were used for reverse transcription (MMLV-RT, Invitrogen) with anchored oligo(dT)23. Quantitative real-time PCR was performed with the LightCycler®480 system using the LightCycler®480 SYBR Green I Master 2X (Roche Applied Science) and gene specific primers listed in Additional file 4: Table S3. Primer specificity and efficiency was confirmed by standard and melting curve analyses. Relative transcript levels (RTL) were calculated relative to the transcript level of the reference gene *TUB6* (At5g12250) as follows: RTL = 1000*2^-(Cptarget-CpTUB6)^. Whole-genome transcriptome analysis was conducted by hybridizing three biological replicate samples of total RNA to Affymetrix GeneChip Arabidopsis ATH1 Genome arrays (Affymetrix, Santa Clara, CA, USA). All steps were conducted at the Nottingham Arabidopsis Stock Centre. Gene expression data were analysed using Partek Genomics Suite 6.6 software (Partek Incorporated, St Louis, USA). The raw CEL files were normalized using the RMA background correction with quantile normalization, log base 2 transformation and mean probe-set summarization with adjustment for GC content. Differentially expressed genes (DEG) were identified by a two-way ANOVA, and *P*-values were adjusted using the FDR (false-discovery rate) method to correct for multiple comparisons. DEG were considered significant if *P*-value was ≤ 0.05 at a fold-change (FC) of ≥ 2 with an FDR < 0.05. Hierarchical clustering in was performed using the default settings in Partek. The average distance between all pairs of objects in the two different clusters was used as the measure of distance between the two clusters, and was measured using Un-weighted Pair-Group Method using arithmetic Averages. Clusters were then merged (agglomerated) until all of the data (genes) were in one cluster.

### Availablity of supporting data

Microarray data are available in the ArrayExpress database (www.ebi.ac.uk/arrayexpress) under accession number E-MTAB-2297.

## Additional files

Additional file 1: Figure S1. Scatter plots of Principle Components 1, 2 and 3 identified from FTIR measurements of cell wall composition. **Figure S2.** JA responsive genes are not up- regulated in *hsr8*. Quantitative Real-time PCR analysis of *VSP1, VSP2* and *ERF1* mRNA levels in Col and *hsr8.* Seedlings were grown vertically in the dark for 14 days on MS medium in the presence of 1 % glucose. Errors bars represent SD from three biological replicates. **, *p* < 0.01 comparing Col to *hsr8-1* (Student’s *t*- test). **Figure S3.** Sugar- regulated gene expression in the single mutant *pft1-2*. (A) to (E) Quantitative Real-time PCR analysis of mRNA levels of the glucose-responsive genes *APL3*, *BAM, GBSS1, GPT2, PDC1* in Col and *pft1-2* in response to glucose*.* Seedlings were grown on MS medium supplemented with 0.5 % glucose in constant light. After 7 days, the seedlings were transferred for 24 h to glucose-free MS liquid medium (solid bars) and then treated for 6 h with 3 % glucose (dashed bars). Errors bars represent SD from three biological replicates. Data shown is representative of three independent experiments. **, *p* < 0.01 comparing glucose responses in Col to *pft1-2* (Student’s *t*- test). (G) and (H) Quantitative Real-time PCR analysis of mRNA levels of the anthocyanin biosynthesis genes *CHS, TT6 and FLS* in Col and *pft1-2* in response to glucose*.* Seedlings were grown on MS medium supplemented with 0.5 % glucose in constant light. After 7 days, the seedlings were transferred for 24 h to glucose-free MS liquid medium (solid bars) and then treated for 6 h with 3 % glucose (dashed bars). Errors bars represent SD from three biological replicates. Data shown is representative of three independent experiments. **, *p* < 0.01 comparing glucose responses in Col to *pft1-2* (Student’s *t*- test). Relative transcript levels (RTL) were calculated relative to the transcript level of the reference gene *TUB6* (At5g12250). (I) Anthocyanin accumulation in response to glucose in Col and *pft1-2* in response to glucose. Seedlings were grown in continuous light for 7 days on MS containing 1 % glucose (solid bars) or 3 % glucose (dashed bars). Errors bars represent SD from three biological replicates. **, *p* < 0.01 comparing glucose responses in Col to *pft1-2* (Student’s *t*- test). Data shown is representative of two independent experiments. **Figure S4.** *PFT1* regulates the expression of genes encoding cell wall- related genes. Quantitative Real-time PCR analysis of genes encoding the pectin methylesterases *PME17* (A), *PME41* (B), and the extensin proteins *AtEXT3* (C) and *AtEXT4* (D) in Col, *hsr8-1*, *pft1-2hsr8-1* and *pft1-2*. Seedlings were grown vertically in the dark for 14 days on MS medium in the presence of 1 % Glucose. Errors bars represent SD from three biological replicates. Panels A-D **, *p* < 0.01 comparing Col to *hsr8-1* (Student’s *t*- test); panels B and D **, *p* < 0.01 comparing *pft1-2 hsr8-1* to *pft1-2* (Student’s *t*- test). Relative transcript levels (RTL) were calculated using transcript levels of the reference gene *TUB6* (At5g12250). (PPTX 97 kb) Additional file 2:
**ANOVA tables of glucose- responsive gene expression in Col and** ***pft1-2*** **.** (XLS 1314 kb) Additional file 3:
**ANOVA tables of gene expression in dark grown seedlings of Col,** ***hsr8-1*** **,** ***hsr8-1pft1-2*** **and** ***pft1-2*** **.** (XLS 68 kb) Additional file 4:
**Primers used in gene amplification and Q-RTPCR.** (DOCX 100 kb)

## Abbreviations

ANOVA: Analysis of variance
ALOG: Arabidopsis LSH1 and oryza G1
*BAM*: β-amylase
*COR*: Cold regulated gene
FTIR: Fourier transform infraRed spectroscopy
GSL: Glucosinolate
JA: Jasmonic Acid
LTP: Lipid Transfer Protein
ᅟ: MED25/PFT1 MEDIATOR25/PHYTOCHROME AND FLOWERING TIME 1
PCA: Principal Component Analysis
*PRL1*: *Pleitropic Regulatory Locus 1*
WAK: Cell Wall-Associated Kinase
