# Supplementary material for: The Mediator complex subunits MED25/PFT1 and MED8 are required for transcriptional responses to changes in cell wall arabinose composition and glucose treatment in Arabidopsis thaliana
Source: BMC Plant Biol. 2015 Sep 5;15:215. doi: 10.1186/s12870-015-0592-4 (PMC4560864; doi:10.1186/s12870-015-0592-4)
Supplement: Additional file 1: Figure S1. — Scatter plots of Principle Components 1, 2 and 3 identified from FTIR measurements of cell wall composition. Figure S2. JA responsive genes are not up- regulated in hsr8. Quantitative Real-time PCR analysis of VSP1, VSP2 and ERF1 mRNA levels in Col and hsr8. Seedlings were grown vertically in the dark for 14 days on MS medium in the presence of 1 % glucose. Errors bars represent SD from three biological replicates. **, p < 0.01 comparing Col to hsr8-1 (Student’s t- test). Figure S3. Sugar- regulated gene expression in the single mutant pft1-2. (A) to (E) Quantitative Real-time PCR analysis of mRNA levels of the glucose-responsive genes APL3, BAM, GBSS1, GPT2, PDC1 in Col and pft1-2 in response to glucose. Seedlings were grown on MS medium supplemented with 0.5 % glucose in constant light. After 7 days, the seedlings were transferred for 24 h to glucose-free MS liquid medium (solid bars) and then treated for 6 h with 3 % glucose (dashed bars). Errors bars represent SD from three biological replicates. Data shown is representative of three independent experiments. **, p < 0.01 comparing glucose responses in Col to pft1-2 (Student’s t- test). (G) and (H) Quantitative Real-time PCR analysis of mRNA levels of the anthocyanin biosynthesis genes CHS, TT6 and FLS in Col and pft1-2 in response to glucose. Seedlings were grown on MS medium supplemented with 0.5 % glucose in constant light. After 7 days, the seedlings were transferred for 24 h to glucose-free MS liquid medium (solid bars) and then treated for 6 h with 3 % glucose (dashed bars). Errors bars represent SD from three biological replicates. Data shown is representative of three independent experiments. **, p < 0.01 comparing glucose responses in Col to pft1-2 (Student’s t- test). Relative transcript levels (RTL) were calculated relative to the transcript level of the reference gene TUB6 (At5g12250). (I) Anthocyanin accumulation in response to glucose in Col and pft1-2 in response to glucose. Seedlings were grown in [file 12870_2015_592_MOESM1_ESM.pptx]

## Slide 1
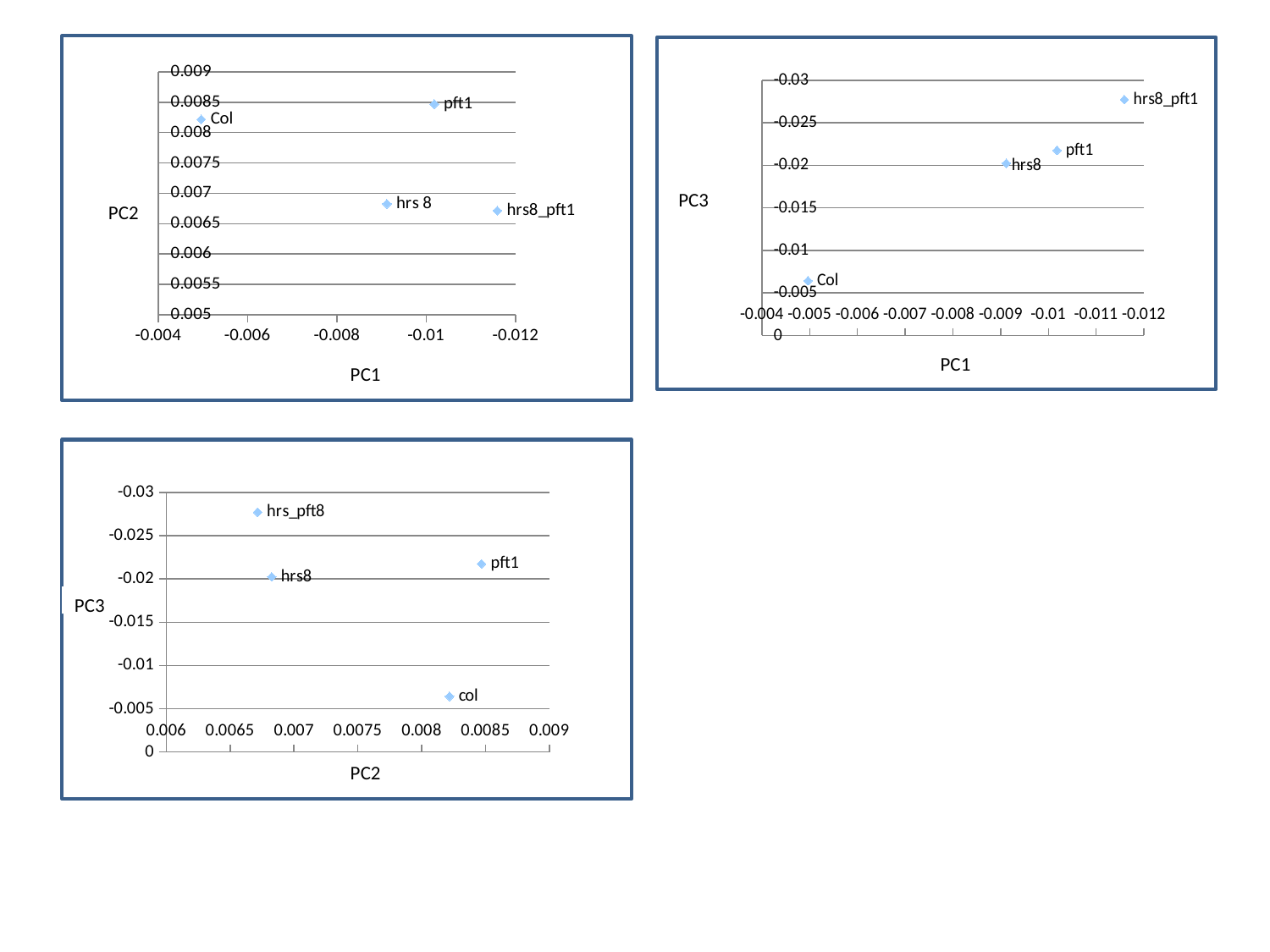

### Chart
| Category | |
|---|---|PC2
PC1
### Chart
| Category | |
|---|---|PC3
PC1
### Chart
| Category | |
|---|---|PC3
PC2

## Slide 2
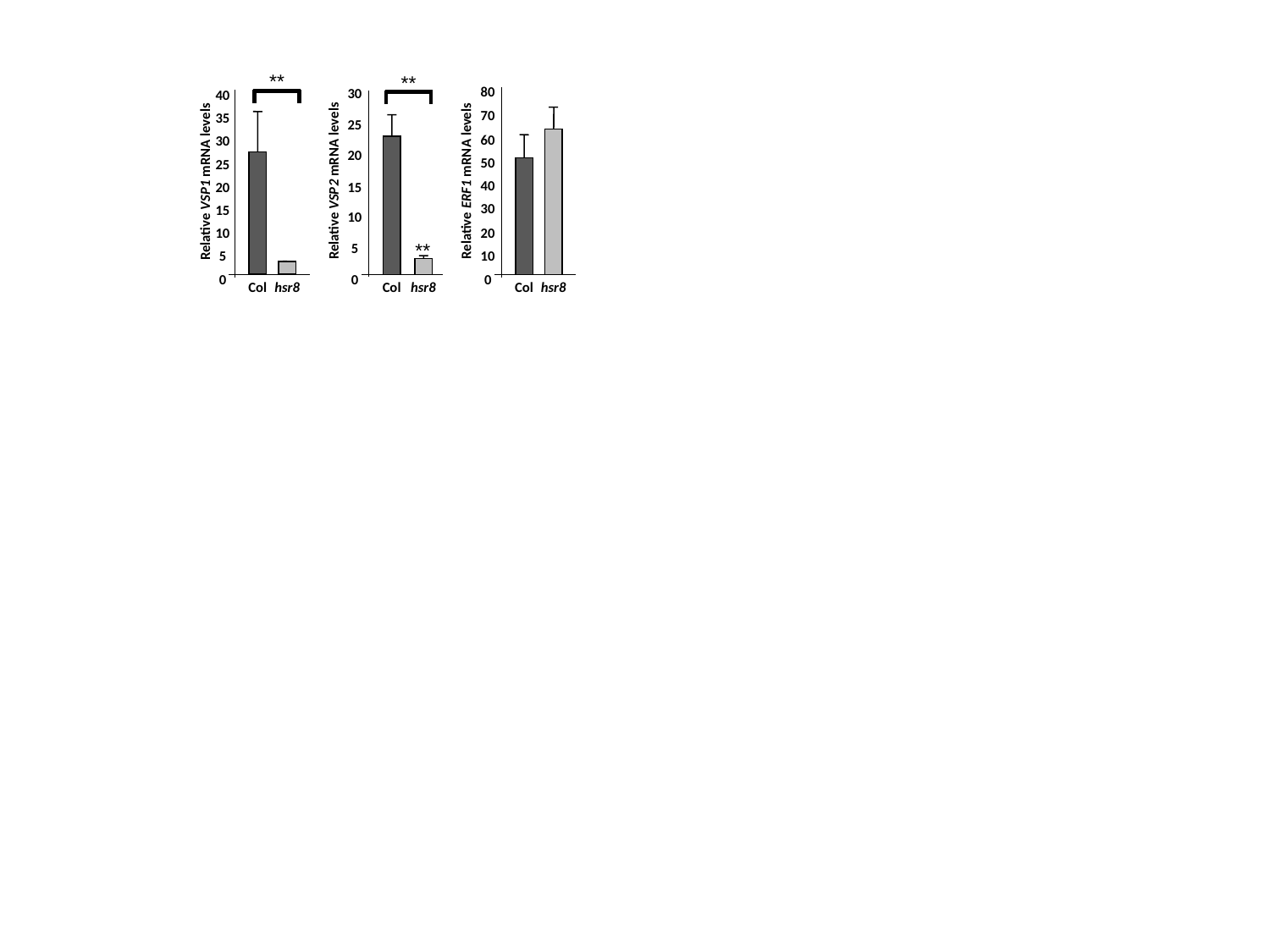

**
**
80
70
60
50
Relative ERF1 mRNA levels
40
30
20
10
0
Col
hsr8
30
25
20
Relative VSP2 mRNA levels
15
10
5
0
Col
hsr8
40
35
30
25
Relative VSP1 mRNA levels
20
15
10
5
0
Col
hsr8
**

## Slide 3
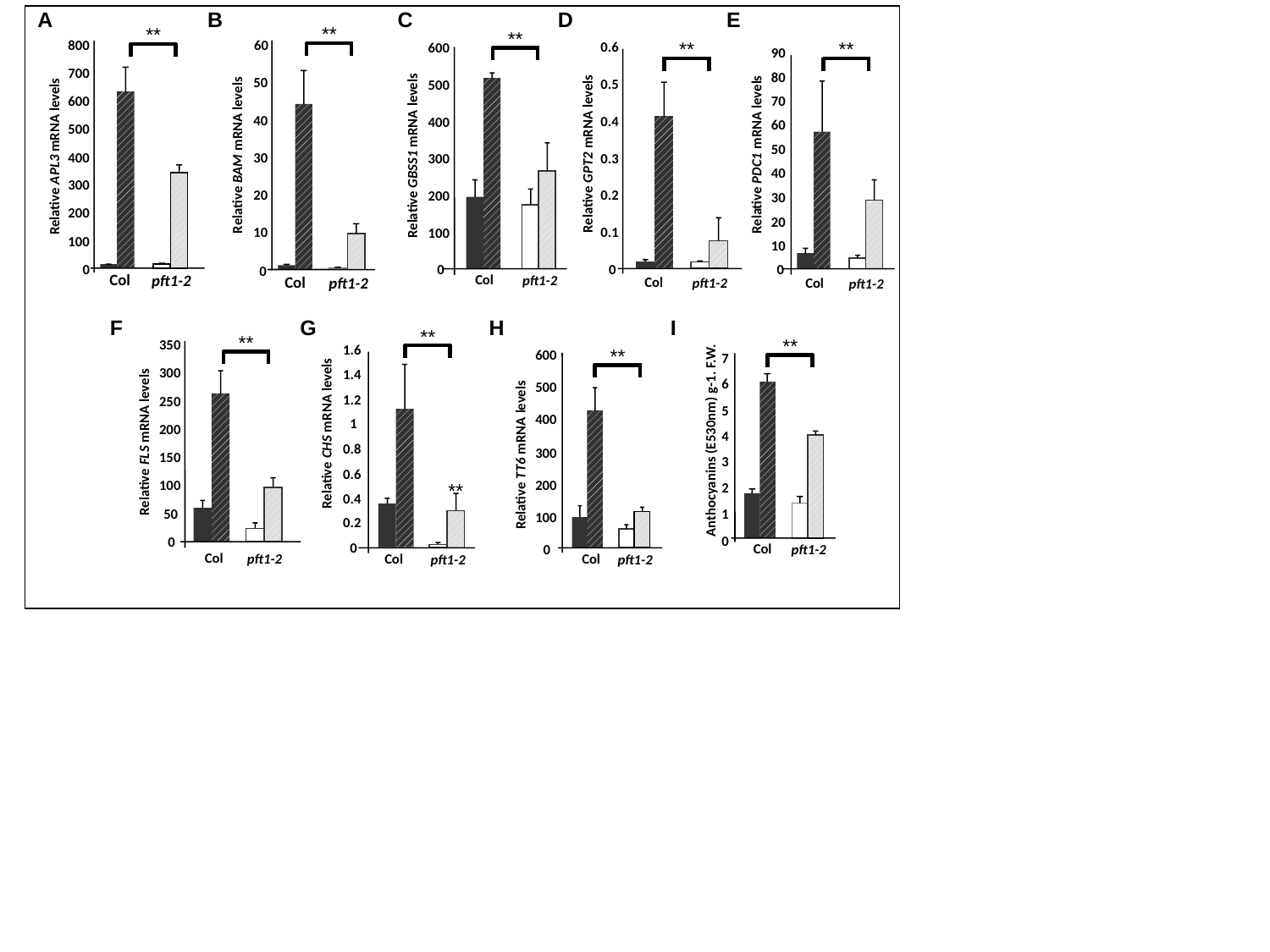

A
B
C
D
E
**
**
**
**
**
800
700
600
500
Relative APL3 mRNA levels
400
300
200
100
0
Col
pft1-2
60
50
40
Relative BAM mRNA levels
30
20
10
0
Col
pft1-2
0.6
0.5
0.4
Relative GPT2 mRNA levels
0.3
0.2
0.1
0
Col
pft1-2
600
500
400
Relative GBSS1 mRNA levels
300
200
100
0
Col
pft1-2
90
80
70
60
50
Relative PDC1 mRNA levels
40
30
20
10
0
Col
pft1-2
F
G
H
I
7
6
5
Anthocyanins (E530nm) g-1. F.W.
4
3
2
1
0
Col
pft1-2
**
**
**
350
300
250
200
Relative FLS mRNA levels
150
100
50
0
Col
pft1-2
**
1.6
1.4
1.2
1
0.8
0.6
0.4
0.2
0
Relative CHS mRNA levels
Col
pft1-2
600
500
400
300
Relative TT6 mRNA levels
**
200
100
0
Col
pft1-2

## Slide 4
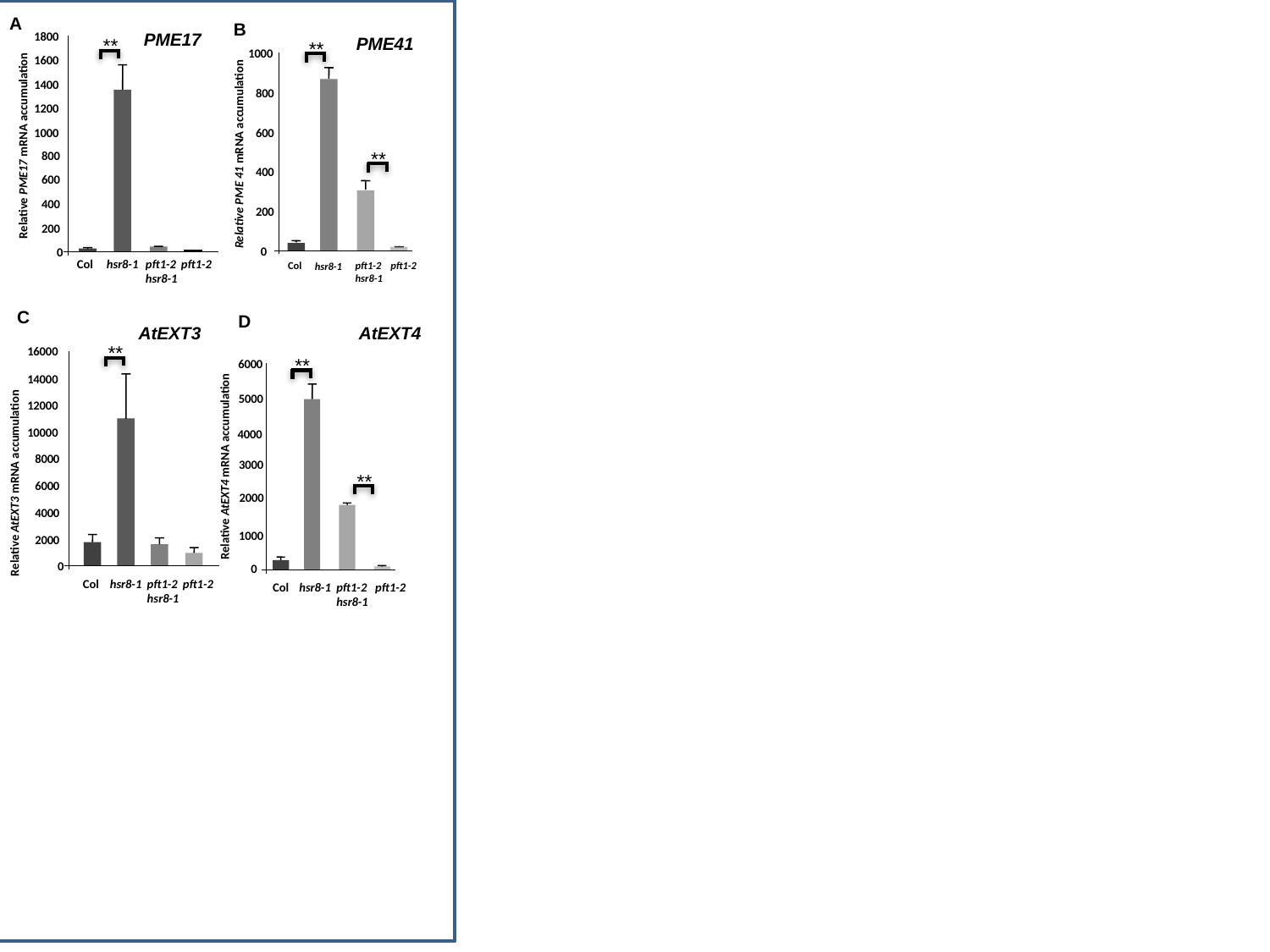

A
B
PME17
PME41
**
1800
1600
1400
1200
1000
Relative PME17 mRNA accumulation
800
600
400
200
0
pft1-2
Col
hsr8-1
pft1-2
hsr8-1
**
1000
800
600
Relative PME 41 mRNA accumulation
400
200
0
Col
pft1-2
hsr8-1
pft1-2
hsr8-1
**
C
D
AtEXT3
AtEXT4
**
16000
14000
12000
10000
8000
Relative AtEXT3 mRNA accumulation
6000
4000
2000
0
pft1-2
Col
pft1-2
hsr8-1
hsr8-1
**
6000
5000
4000
3000
Relative AtEXT4 mRNA accumulation
2000
1000
0
Col
hsr8-1
pft1-2
hsr8-1
pft1-2
**
