# Supplementary material for: The Mediator complex subunits MED25/PFT1 and MED8 are required for transcriptional responses to changes in cell wall arabinose composition and glucose treatment in Arabidopsis thaliana
Source: BMC Plant Biol. 2015 Sep 5;15:215. doi: 10.1186/s12870-015-0592-4 (PMC4560864; doi:10.1186/s12870-015-0592-4)
Supplement: Additional file 4: — Primers used in gene amplification and Q-RTPCR. (DOCX 100 kb) [file 12870_2015_592_MOESM4_ESM.docx]

**Table S3**. Primers used for genomic complementation and Quantitative Real-Time PCR.

| Genomic complementation |  |
| --- | --- |
| at1g25510gfor | ttttgggcccgcagtgaaacttacacaactccacc |
| at1g25510grev | aaaacccggggttagtggagtcactcatgatgg |
| at1g25520gfor | ttttgggcccgacgcgtgtgaagtactcgccac |
| at1g25520grev | aaaacccgggagcttggctcattgctgctgc |
| at1g25530gfor | ttttgggcccaacagcagcagcaacaacaactc |
| at1g25530grev | aaaacccgggagactgcaagtggaaattgcctg |
| at1g25540gfor | ttttgggcccgtcaccgctcttgaacatcatcat |
| at1g25540grev | aaaacccgggagcaatatccaacgtgagaagtgag |
| at1g25550gfor | ttttgggcccctctccatttaactcactgccacag |
| at1g25550grev | aaaacccggggcttggtttactaagggaaaggctg |
| at1g25560gfor | ttttgggccctatgaggaggatggtgctgagtg |
| at1g25560grev | aaaacccgggtgggctccaagtcatgagaattg |
|  |  |
| Quantitative Real-Time PCR |  |
| qPCR TUB6 for | ggtgaaggaatggacgagat |
| qPCR TUB6 rev | gtcatctgcagttgcgtctt |
| qPCR βPCR  for | gtactgagcaaggcatggaa |
| qPCR βPCR gag rev | gcctcgcgttaagaagaatc |
| qPCR APL3 for | gttaaggaggctgataggccagagg |
| qPCR APL3 rev | caccacggcggtcttgagatcg |
| qPCR PDF1.2 for | tttgctgctttcgacgcac |
| qPCR PDF1.2 rev | cgcaaacccctgaccatg |
| qPCR VSP2 for | tcagtgaccgttggaagttgtg |
| qPCR VSP2 rev | gttcgaaccattaggcttcaatatg |
| qPCR MYC2 for | tcatacgacggttgccagaa |
| qPCR MYC2 rev | agcaacgtttacaagctttgattg |
| qPCR ERF1 for | cgagaagctcgggtggtagt |
| qPCR ERF1 rev | gccgtgcatccttttcc |
| qPCR at1g32900 for | ggtcaagaactgcatggaccaag |
| qPCR at1g32900 rev | cgtcgctacgttctccttggcc |
| qPCR at1g61800 for | tcttcgtcttccttctccacc |
| qPCR at1g61800 rev | aacacactttgtgccactacc |
| qPCR at4g33070 for | gaagcaacacagaatcttcaatgc |
| qPCR at4g33070 rev | gaaacatgattaaacccaacggtcg |
| qPCR FLS for | ggacgacggtggataaagag |
| qPCR FLS rev | tccggttagttccggtaaag |
| qPCR CHS for | cctcaaggagaagttcaagcg |
| qPCR CHS rev | aacgctgtgcaagacgactg |
| qPCR TT6 for | taaagaggagcgtgaccaca |
| qPCR TT6 rev | aacagaaccaacgcaacaac |
| qPCR ASN1for | ttccctcacaacactccaaa |
| qPCR ASN1rev | cacctccaggaaccgttagt |
| qPCR at1g19000 for | aaacacagatgctgctccac |
| qPCR at1g19000 rev | gtgaaagccgagtgtcttga |
| qPCR at5g47390 for | agaccaactgctgtgcactc |
| qPCR at5g47390 rev | cggattctccatgtttgttg |
